# Spinal anesthesia for cesarean section in a woman with an intrathecal baclofen pump

**DOI:** 10.1186/s40981-022-00515-6

**Published:** 2022-03-22

**Authors:** Heath Allen, Ryu Komatsu, Hani El-Omrani

**Affiliations:** 1 Denali Anesthesia, Alaska Regional Hospital, 2801 Debarr Road, Anchorage, AK 99508 USA; 2 Department of Anesthesiology and Pain Medicine, University of Washington, Box 356540, 1959 NE Pacific Street, BB-1469, Seattle, WA 98195-6540 USA

**Keywords:** Intrathecal pump, Baclofen, Neuraxial anesthesia, Cesarean section

## Abstract

**Background:**

Intrathecal baclofen pumps are commonly used for the management of lower extremity spasticity in the setting of spinal cord injury. There have been no reports of the performance of spinal anesthesia in patients with a pre-existing intrathecal baclofen pump.

**Case presentation:**

A 29-year-old parturient presented for cesarean section. She had a history of spinal cord injury due to fractures of the thoracic vertebrae with lower extremity spasticity, which had been treated with an intrathecal baclofen pump inserted through lumbar (L) 3-L4 intervertebral space. Preoperative lumbosacral ultrasound was performed to identify the L4-5 interspace, and spinal anesthesia was performed through that space with a 25-gauge 3.5-inch-long Whitacre spinal needle. Thoracic (T) 4 dermatomal level anesthesia was achieved, and the patient underwent the cesarean section without requiring additional intravenous analgesic adjuncts.

**Conclusions:**

Spinal anesthesia can be successfully performed in patients with intrathecal baclofen pumps. Existing intrathecal catheters can be located with preoperative imaging, and ultrasound can be used to determine the vertebral levels below the intrathecal catheter through which spinal anesthesia can be performed safely.

## Background

**Fig. 1:**
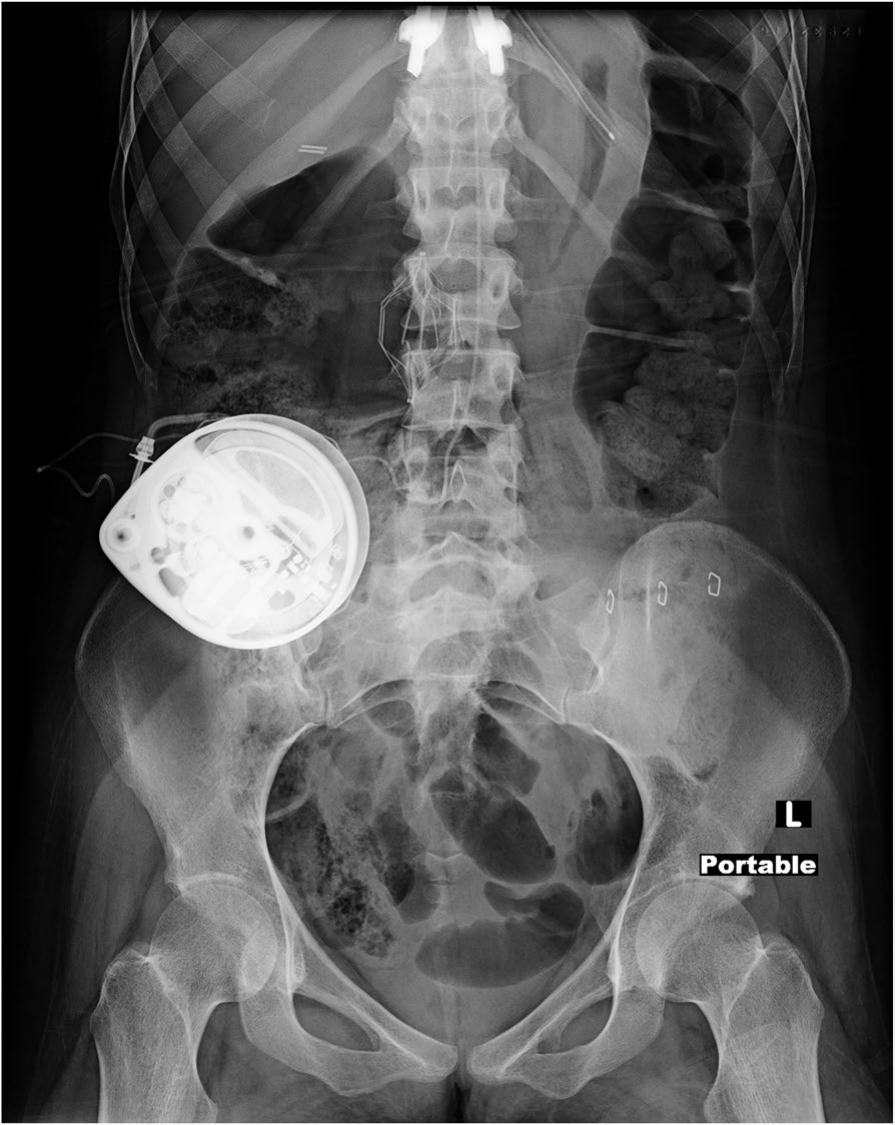
Plain abdominal x-ray showing baclofen pump with tubing present intrathecally

Intrathecal baclofen pumps are commonly used for the management of lower extremity spasticity in the setting of spinal cord injury. The American Society of Regional Anesthesia and Pain Medicine (ASRA) recommends the use of regional anesthesia in patients with pre-existing intrathecal baclofen pumps whenever possible. In the setting of obstetric anesthesia with a known catheter location, it further recommends placing the needle one to two intervertebral spaces caudad to the intrathecal catheter entry point [1]. In spite of this, there are currently no case studies in the literature of spinal anesthesia in the setting of an intrathecal baclofen pump, and many anesthesiologists are reluctant to employ neuraxial anesthetics in this setting. In this case report, we describe the successful use of spinal anesthesia for cesarean section in a patient with an intrathecal baclofen pump (Fig. 1).

## Case presentation

The written informed consent was obtained from the patient for publication of this care report. This study was conducted with an Institutional Review Board (IRB) waiver (University of Washington IRB). A 29-year-old gravida 2 para 0 woman at 36 weeks’ gestation presented for elective cesarean section. Maternal history included incomplete paraplegia at thoracic (T) 6-T7 with traumatic brain injury and fractures of the thoracic vertebrae secondary to a motor vehicle accident at age 13. She had related T2-T10 spinal fixation with rods, lower extremity spasticity, and neurogenic bladder but no sensory loss and no history of autonomic dysreflexia. She was able to walk short distances but often used a wheelchair due to weakness. Her lower extremity spasticity was managed by an intrathecal baclofen pump. The pump reservoir was located subcutaneously in the right lower quadrant and a previously obtained abdominal x-ray showed the catheter traversing medially and entering the intrathecal space on the right of midline between the lumbar (L) 3 and L4 spinous processes and the tip of the catheter at the level of thoracic (T) 10 and T11 interspace in the midline. She also had a history of complex rectovaginal laceration status-post vaginal repair, with diverting colostomy and subsequent colostomy take down as well as a laparoscopic cholecystectomy. This history necessitated a cesarean section and predicted longer than normal operative times. The neurosurgeon who had placed the pump suggested neuraxial anesthesia placement on the left side L2-3 or L4-5. After discussing the risks and benefits of general versus regional anesthesia, the decision was made to proceed with spinal anesthesia.

Preoperative lumbosacral ultrasound was performed to identify the L4-5 interspace with a dural depth of approximately 5cm. After positioning the patient in the sitting position, her back was prepped and draped and her skin was localized with 1% lidocaine. A 20-gauge 1.25-inch-long introducer needle was inserted using a left paramedian approach at the L4-5 interspace. A 25-gauge 3.5-inch-long Whitacre spinal needle was inserted through the introducer and cerebrospinal fluid flowed freely from the needle. There were no difficulties with placement or paresthesia. Cerebrospinal fluid was aspirated and a mixture of 1.8 ml of 0.75% hyperbaric bupivacaine, 10 mcg fentanyl, 100 mcg morphine, 100 mcg epinephrine, and 25 mcg clonidine were injected into the intrathecal space. Blood pressure was measured with NIBP taken every minute after spinal with phenylephrine infusion initiated immediately with the spinal dose and titrated to maintain normotension.

After 5 min of spinal anesthesia placement, pinprick testing showed a bilateral T7 level block height which progressed to T4 level bilaterally after 15 min. The patient was comfortable for the entire 94-min procedure and no intravenous analgesic adjuncts were required. The baby was delivered uneventfully at a weight of 2.4 kg with an Apgar score 9 at 1 min and 9 at 5 min. Postoperatively, her sensation and lower extremity strength returned to baseline. Her postoperative pain was controlled with oral ibuprofen and acetaminophen. Her spasticity postoperatively was improved from baseline.

## Discussion

We were able to find two case reports of epidural anesthesia for vaginal delivery [2, 3], and one for postoperative pain management in patients with intrathecal baclofen pumps [4], but found no reports of spinal anesthesia in patients with baclofen pumps.

We identified multiple considerations related to spinal anesthesia in the setting of an intrathecal baclofen pump. First is the potential for damaging the intrathecal catheter. This would require surgical replacement as well as management of potentially life-threatening baclofen withdrawal. We were able to minimize this risk by identifying the location of the catheter on x-ray, and the corresponding intervertebral space with ultrasound. Second, there is a theoretical concern of introducing bacteria to the intrathecal catheter. This risk was mitigated with the use of sterile technique during spinal anesthesia placement. Third, there is a theoretical concern of drug interaction between the intrathecal baclofen and spinal medication. This concern is minimal given that there are no reports in the literature describing any interactions between baclofen and the drugs used in the spinal anesthetic. The baclofen catheter tip is conventionally placed in the thoracic region and the volume infused is commonly a fraction of a milliliter/day (in this patient 177 mcg/day or 0.35 mL/day). Total CSF volume in an average adult is 150 mL, and bulk flow and diffusion of this very small volume of baclofen infusate is unlikely to have an effect on spinal medications.

Epidural placement in the setting of previous spinal procedures carries the risk of patchy block secondary to scar tissue in the epidural space; however, in the setting of spinal anesthesia, this is less likely to be a concern.

For our spinal dose, epinephrine and a larger than typical dose of bupivacaine were chosen to prolong the block in the setting of expected prolonged surgical time. We chose this approach rather than attempting a combined spinal-epidural anesthesia from a concern that scar tissue from previous spinal procedures could disrupt the spread of epidural medication. Clonidine was chosen as a synergistic analgesic adjunct and to treat patient anxiety.

## Conclusions

Spinal anesthesia can be safely and successfully performed in patients with intrathecal baclofen pumps. Pre-existing intrathecal catheters can be located with preoperative imaging and ultrasound can be used to determine a safe injection site below or above the intrathecal catheter.

## Data Availability

Unidentified patient’s information used during the current study is available from the corresponding author on reasonable request. No datasets were formed for the current study.

## Abbreviations

L: Lumbar
T: Thoracic
ASRA: American Society of Regional Anesthesia and Pain Medicine
IRB: Institutional Review Board
CSF: Cerebrospinal fluid
